# The Renewal of Life; Lectures, Chiefly Clinical

**Published:** 1867-03

**Authors:** 


The Renewal of Life; Lectures, Chiefly Clinical. By THOMAS K. CHAMBERS, M.D., Honorary Physician to H. R. H. the Prince of Wales; Consulting Physician and Lecturer on the Practice of Medicine, at St. Mary's Hospital; Consulting Physician to the Lock Hospital. Second American, from the Fourth London Edition. Philadelphia: LINDSAY &

BLAKISTON. 1866. pp. 646. Price $5.00. For sale at S. C. GRIGGS & Co.'s., Lake street.

The subjects discussed in this volume are as follows: Death and Life; Disease and Cure; Formation of Mucus and Pus; Typh. Fever; Small-Pox; Rheumatic Fever; Gonorrhœal Rheumatism; Pericarditis; Pleurisy; Hydrothorax; Acute Laryngitis; Capillary Catarrh; Pneumonia; Emphysema of the Lungs; Pulmonary Consumption; Thoracic Aneurism; Disease of the Heart; Purpura; Anœmia; Prominence of Eyeballs; Atrophy of Muscles; Chorea; Epilepsy; Hysteria; Spinal Paralysis; Sciatica; Albumanuria; Ascites; Diabetis; Mortification; Importance of Digestive Organs in Therapeutics; Indigestion in General; Slow Digestion and Acidity; Pain in the Stomach; Emetation and Vomiting; Diarrhœa; Costiveness and Constipation; Dietetics; Corpulence; On Pepsine; On Alcohol; On Blood-letting; Review; L'Envoi.

This list of important subjects is pretty fully discussed by one of the most interesting and experienced writers of our day. Hence the practitioner will find it a very valuable addition to his library. The book is published in excellent style.

